# Supplementary material for: The Cell-Penetrating Peptide GV1001 Enhances Bone Formation via Pin1-Mediated Augmentation of Runx2 and Osterix Stability
Source: Biomolecules. 2024 Jul 8;14(7):812. doi: 10.3390/biom14070812 (PMC11274716; doi:10.3390/biom14070812)

Supplement Figure . Original western blot for three repeats of Figure 1F

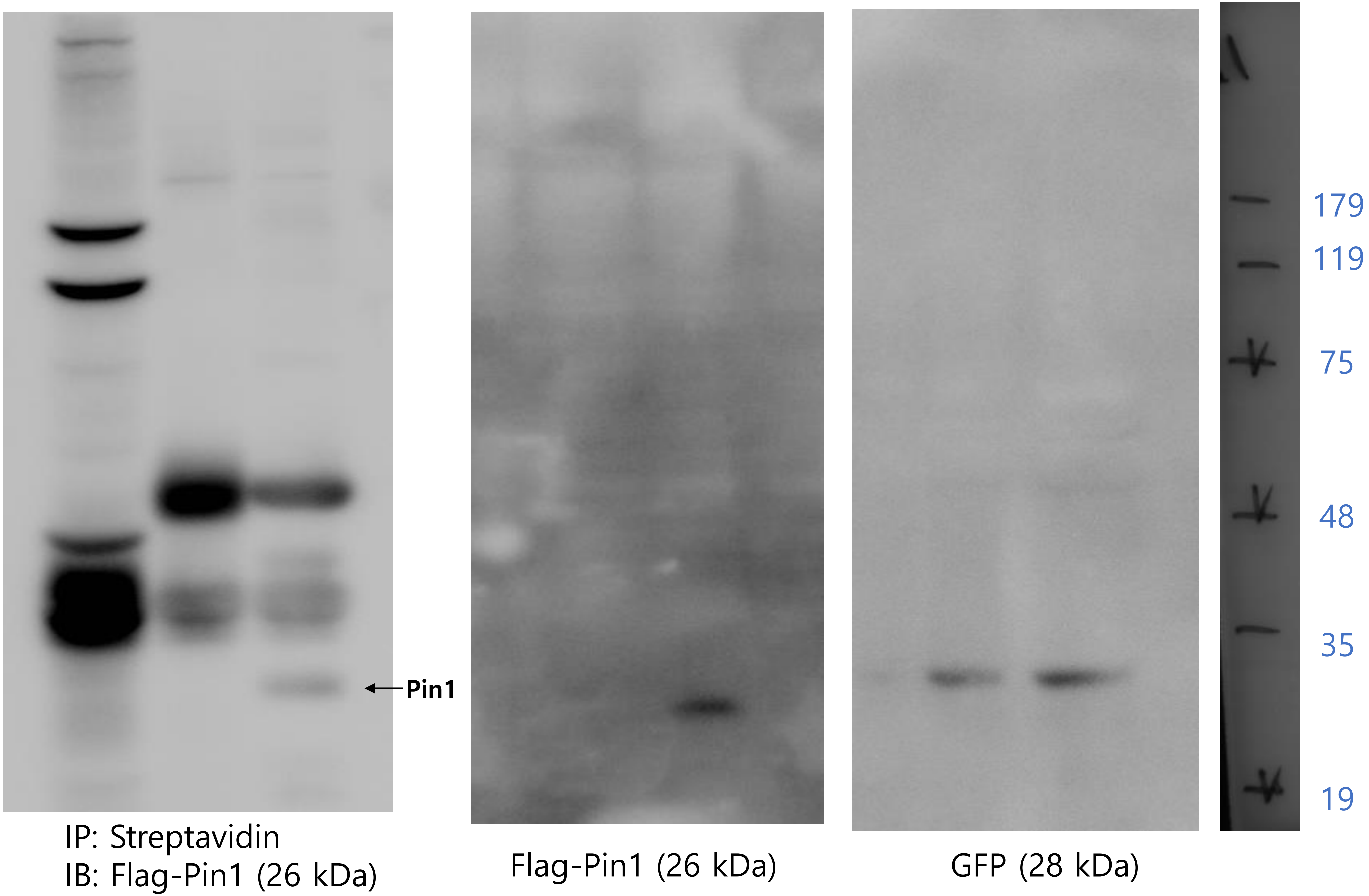

Supplement Figure . Original western blot for three repeats of Figure 2C

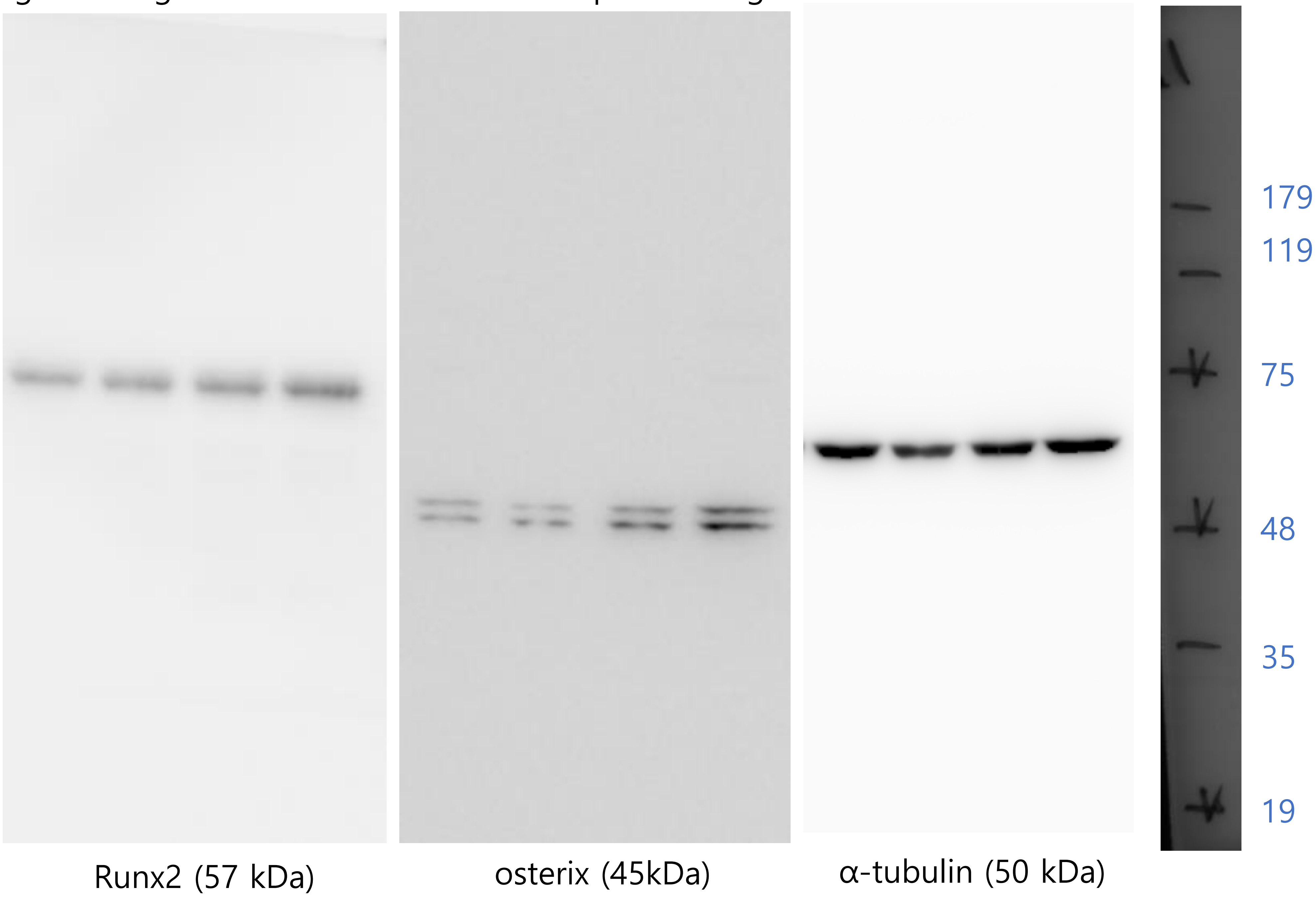

Supplement Figure . Original western blot for three repeats of Figure 3C

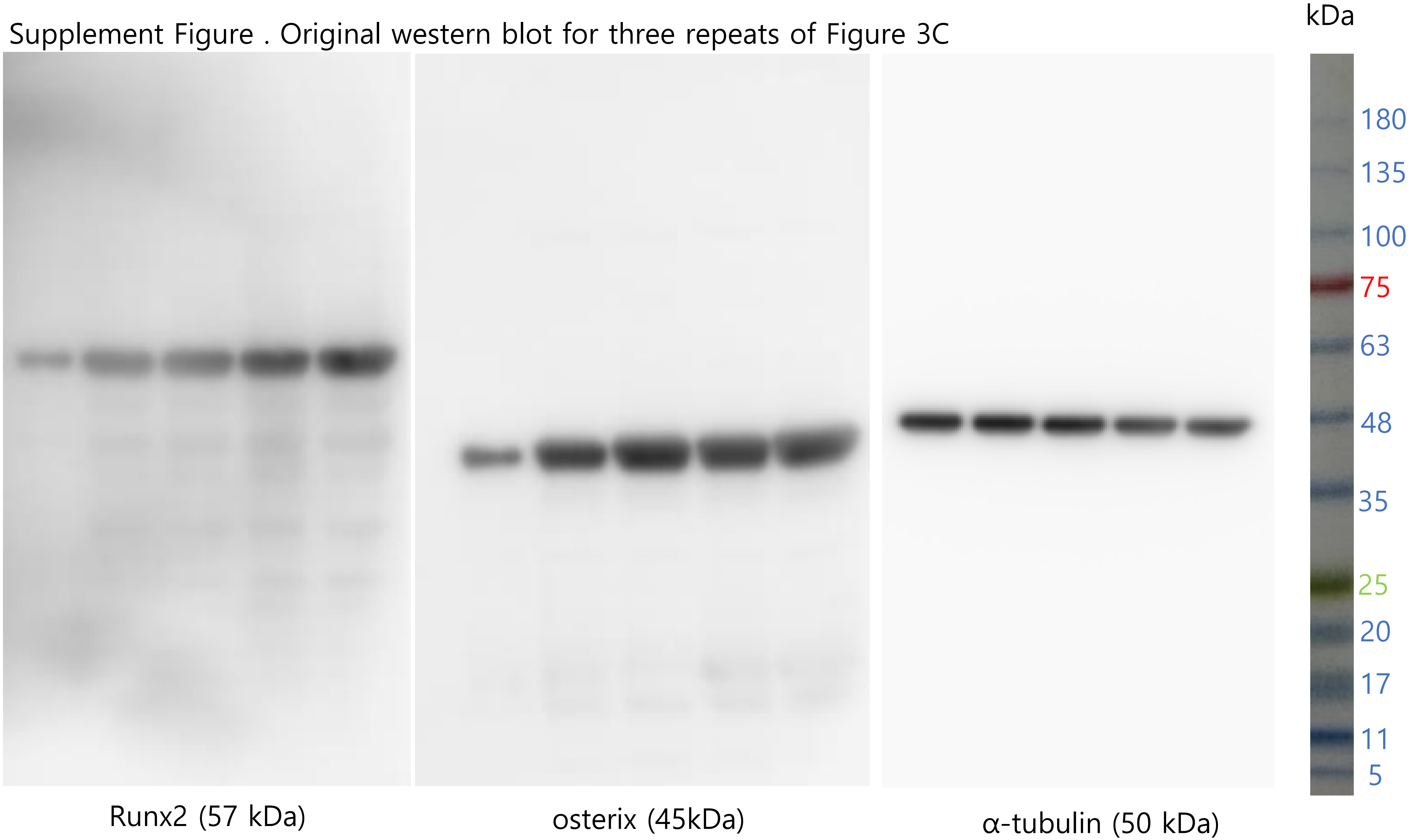

Supplement Figure . Original western blot for three repeats of Figure 4C

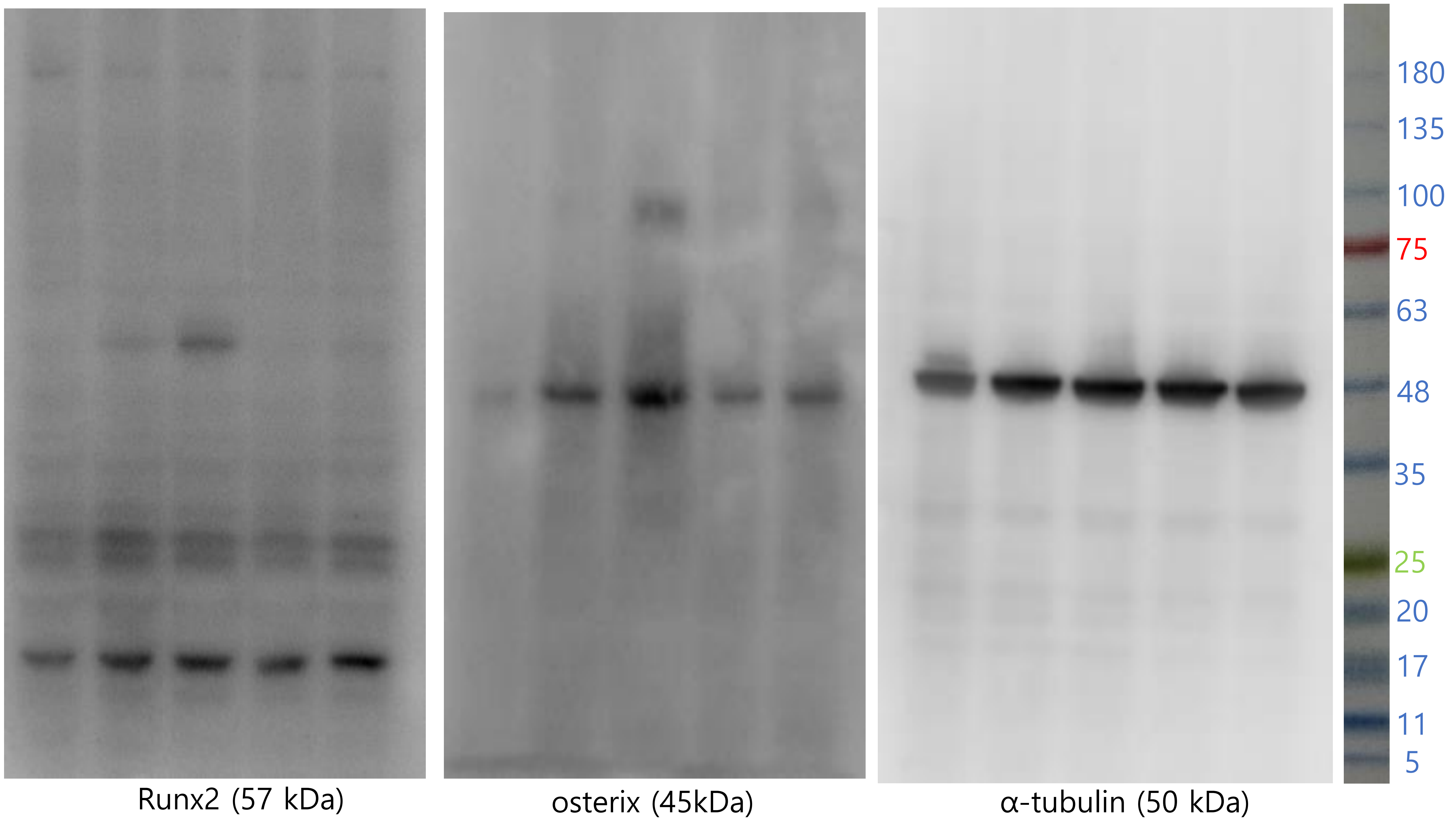

Supplement Figure . Original western blot for three repeats of Figure 5C

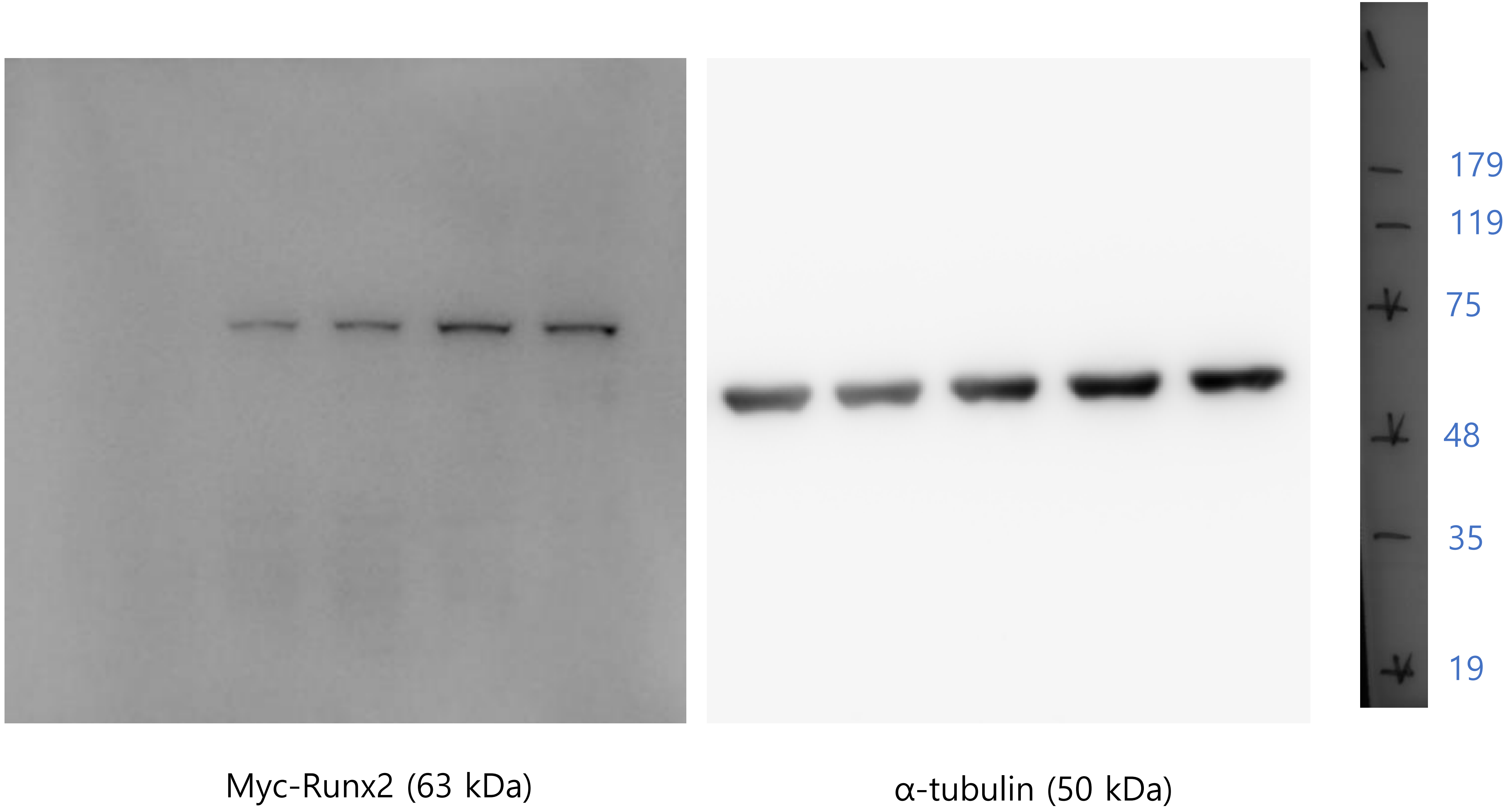

Supplement Figure . Original western blot for three repeats of Figure 5D

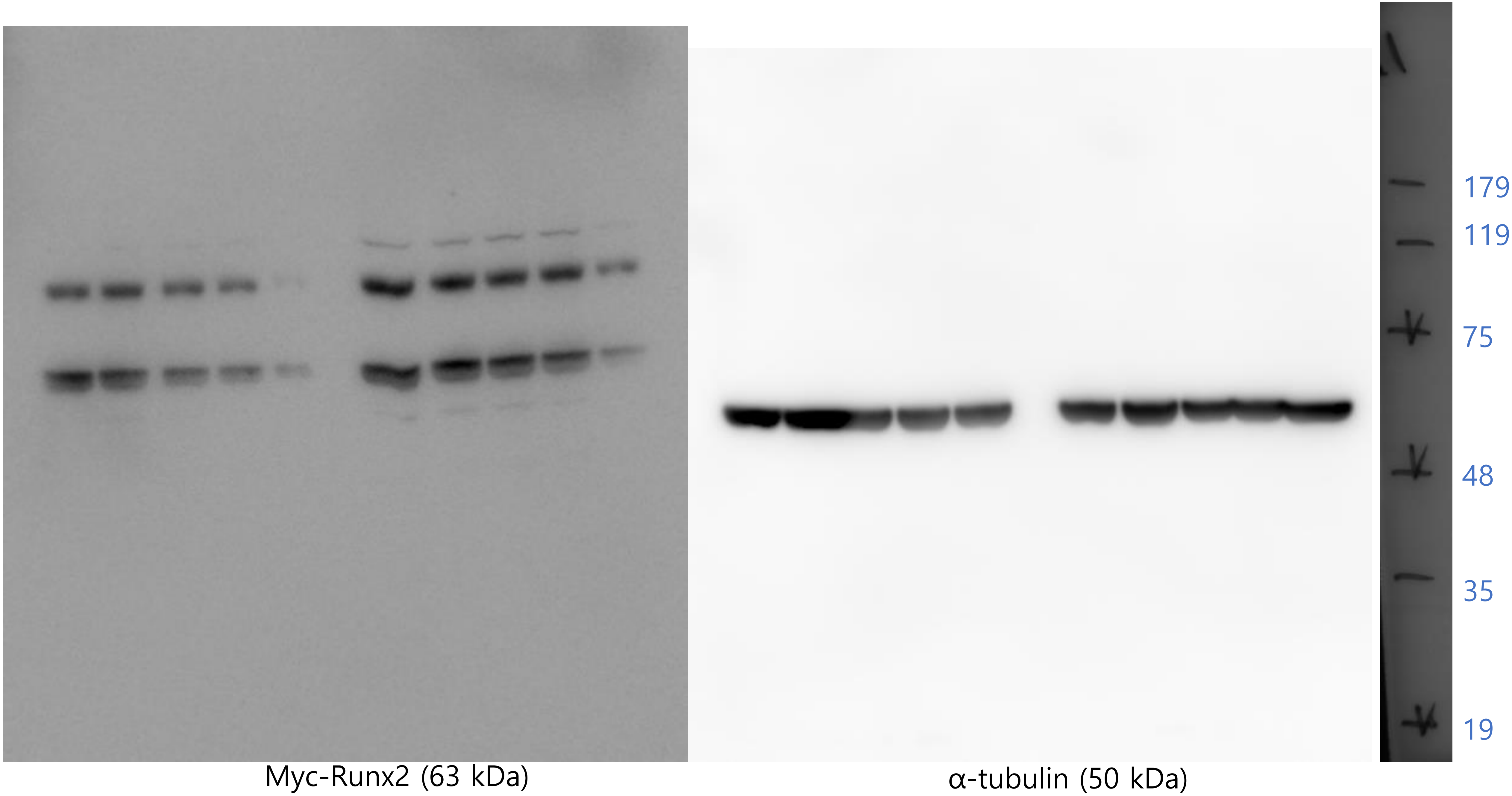

Supplement Figure . Original western blot for three repeats of Figure 6C

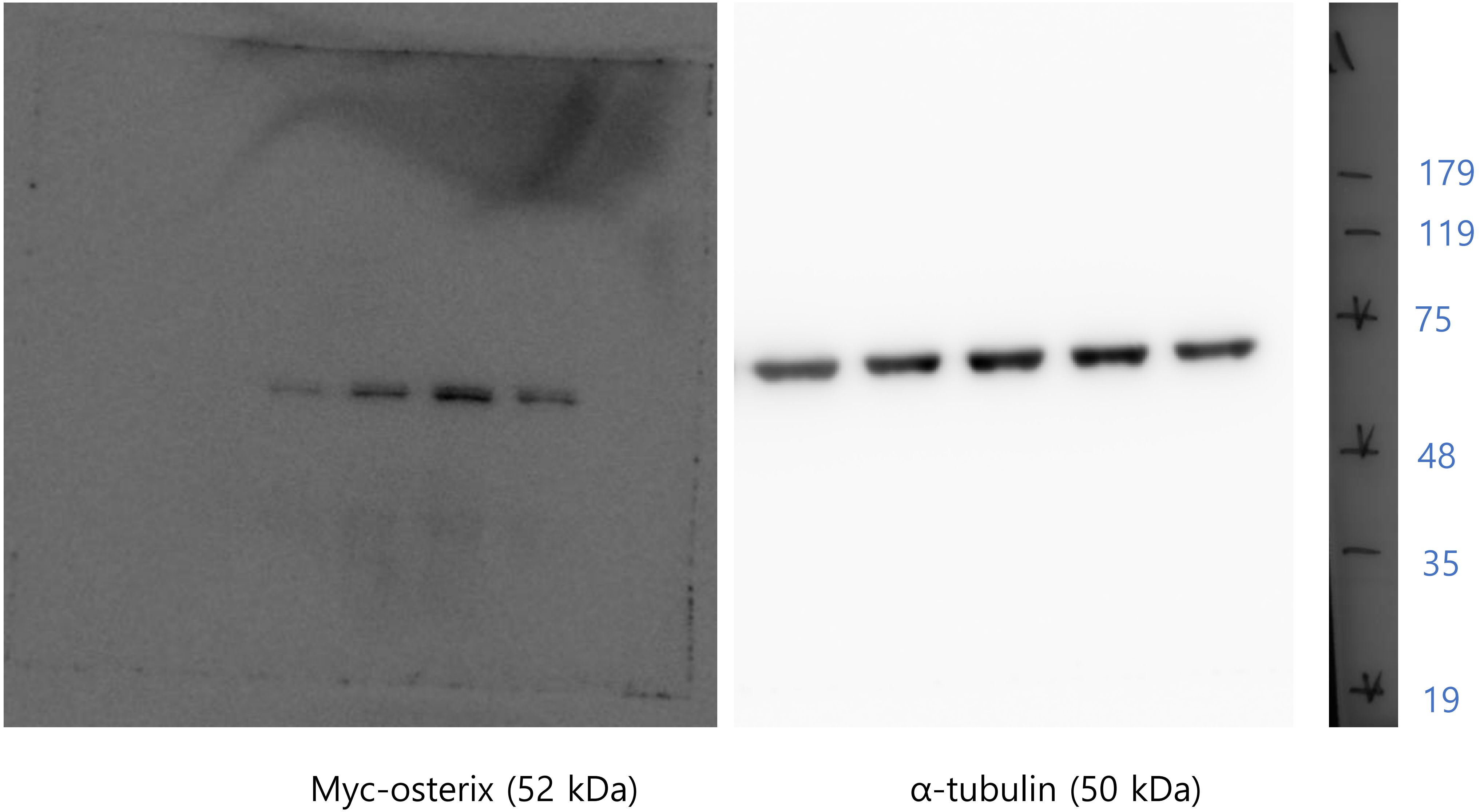

Supplement Figure . Original western blot for three repeats of Figure 6D

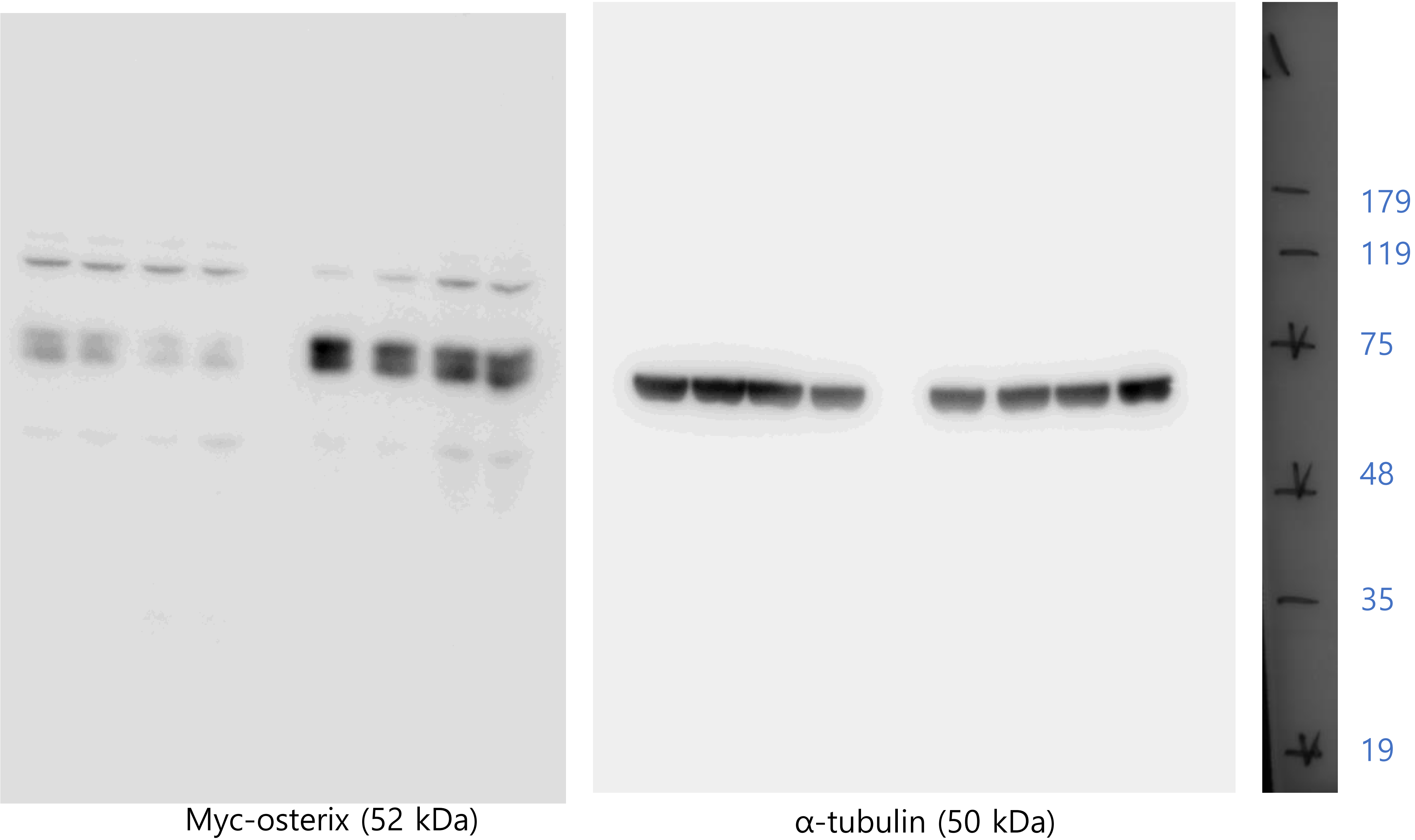

Supplement: Supplementary file 1 [file biomolecules-14-00812-s001.zip › biomolecules-3064742-original-images.pdf]
