# Supplementary material for: The Cell-Penetrating Peptide GV1001 Enhances Bone Formation via Pin1-Mediated Augmentation of Runx2 and Osterix Stability
Source: Biomolecules. 2024 Jul 8;14(7):812. doi: 10.3390/biom14070812 (PMC11274716; doi:10.3390/biom14070812)
Supplement: Supplementary file 1 [file biomolecules-14-00812-s001.zip › biomolecules-3064742-s1.pdf]

SUPPLEMENTAL FIGURES

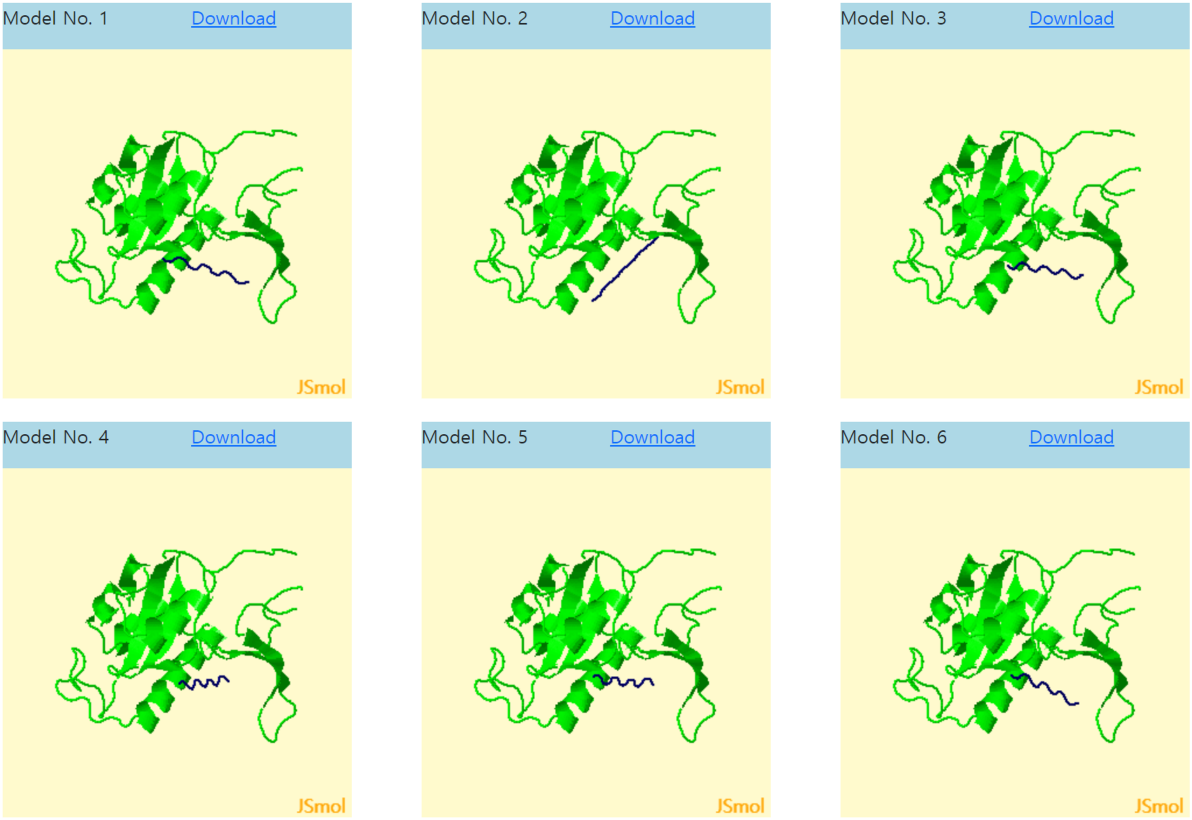

Supplemental Figure S1. Molecular docking mode GV1001 in Pin1 Docking models of GV1001 on Pin1(PDB DOI: https://doi.org/10.2210/pdb1PIN/pdb). The three-dimensional solid ribbon in green represents the structure of Pin1, while the blue ribbon indicates GV1001. We used the GRAMM docking program (https://gramm.compbio.ku.edu/gramm) to dock GV1001 into the crystal structure of Pin1.
